# 583 Risk Factors Associated with Subtherapeutic Enoxaparin Levels in Burn Patients

**DOI:** 10.1093/jbcr/irae036.217

**Published:** 2024-04-17

**Authors:** Desiree Pinto, Sophia Lee, Cory Johnson, Rola A Halabi, Shawn Tejiram, Taryn E Travis, Lauren T Moffatt, Jeffrey W Shupp

**Affiliations:** MedStar Washington Hospital Center, Washington, DC; Georgetown University School of Medicine, Washington, DC; MedStar Washington Hospital Center, Georgetown University School of Medicine, Washington, DC; Firefighters’ Burn and Surgical Research Laboratory, Washington, DC; MedStar Health |Georgetown University School of Medicine, Washington, DC, DC; MedStar Washington Hospital Center, Washington, DC; Georgetown University School of Medicine, Washington, DC; MedStar Washington Hospital Center, Georgetown University School of Medicine, Washington, DC; Firefighters’ Burn and Surgical Research Laboratory, Washington, DC; MedStar Health |Georgetown University School of Medicine, Washington, DC, DC; MedStar Washington Hospital Center, Washington, DC; Georgetown University School of Medicine, Washington, DC; MedStar Washington Hospital Center, Georgetown University School of Medicine, Washington, DC; Firefighters’ Burn and Surgical Research Laboratory, Washington, DC; MedStar Health |Georgetown University School of Medicine, Washington, DC, DC; MedStar Washington Hospital Center, Washington, DC; Georgetown University School of Medicine, Washington, DC; MedStar Washington Hospital Center, Georgetown University School of Medicine, Washington, DC; Firefighters’ Burn and Surgical Research Laboratory, Washington, DC; MedStar Health |Georgetown University School of Medicine, Washington, DC, DC; MedStar Washington Hospital Center, Washington, DC; Georgetown University School of Medicine, Washington, DC; MedStar Washington Hospital Center, Georgetown University School of Medicine, Washington, DC; Firefighters’ Burn and Surgical Research Laboratory, Washington, DC; MedStar Health |Georgetown University School of Medicine, Washington, DC, DC; MedStar Washington Hospital Center, Washington, DC; Georgetown University School of Medicine, Washington, DC; MedStar Washington Hospital Center, Georgetown University School of Medicine, Washington, DC; Firefighters’ Burn and Surgical Research Laboratory, Washington, DC; MedStar Health |Georgetown University School of Medicine, Washington, DC, DC; MedStar Washington Hospital Center, Washington, DC; Georgetown University School of Medicine, Washington, DC; MedStar Washington Hospital Center, Georgetown University School of Medicine, Washington, DC; Firefighters’ Burn and Surgical Research Laboratory, Washington, DC; MedStar Health |Georgetown University School of Medicine, Washington, DC, DC; MedStar Washington Hospital Center, Washington, DC; Georgetown University School of Medicine, Washington, DC; MedStar Washington Hospital Center, Georgetown University School of Medicine, Washington, DC; Firefighters’ Burn and Surgical Research Laboratory, Washington, DC; MedStar Health |Georgetown University School of Medicine, Washington, DC, DC

## Abstract

**Introduction:**

A hypermetabolic state develops after severe burn injury altering volume distribution, creatinine clearance (CrCl), and hepatic metabolism. This ultimately impacts pharmacokinetics of drugs, including enoxaparin, the standard prophylactic venous thromboembolism (VTE) anticoagulant. Understanding specific factors in burn patients that impact the pharmacokinetics of enoxaparin is necessary to reduce rates of VTE. This study aims to identify risk factors associated with subtherapeutic levels of enoxaparin, measured by peak anti-Xa levels, in burn patients.

**Methods:**

Patients admitted to a regional burn center over a six-month period were included in this retrospective review. Patients on enoxaparin 40mg twice a day (BID) for VTE prophylaxis and had a peak anti-Xa level were included. Exclusion criteria included polytrauma, therapeutic anticoagulation or anti-platelet therapy, a history of chronic kidney disease, cirrhosis, coagulation disorder, heparin induced thrombocytopenia (HIT), or thrombocytopenia at admission. Patient demographics, burn injury characteristics, and lab values for peak anti-Xa levels and admission CrCl were obtained. For prophylactic enoxaparin, a therapeutic level is 0.2-0.4U/mL and a subtherapeutic level is < 0.2U/mL. Patients were separated into groups based on therapeutic or subtherapeutic levels. Differences between groups were evaluated using two-sample t-test or Wilcoxon rank-sum test.

**Results:**

Both the subtherapeutic and therapeutic groups had 13 patients. No patient developed a symptomatic VTE within 31 days of discharge. There were no significant differences in gender, BMI (30.4 vs 31.6 kg/m2), presence of inhalation injury, or ICU length of stay (5 vs 3 days) between groups. Patients in the subtherapeutic group were significantly younger (38 vs 48 years), with a higher TBSA (22.6% vs 6%), and a higher CrCl at admission (134.2 vs 104.8 mg/dL). In the subtherapeutic group, the highest dose needed to reach a therapeutic level of enoxaparin was 60mg BID and the median final dose was 50mg BID.

**Conclusions:**

A delay in initiation of VTE prophylaxis is associated with increased rates of VTE in burn patients, and a delay to therapeutic levels of VTE prophylaxis will likely lead to increased rates of VTE as well. Factors associated with a delay to therapeutic enoxaparin levels were age, %TBSA, and CrCl. A uniform standard dose of enoxaparin may not be effective for all burn patients.

**Applicability of Research to Practice:**

Burn patients are a unique subgroup that require special consideration in every aspect of care, including VTE prophylaxis. Risk factors associated with subtherapeutic enoxaparin levels after burn injury need to be identified to guide the development of effective VTE prophylaxis guidelines.

